# The Effect of Isotretinoin on Insulin Resistance and Serum Adiponectin Levels in Acne Vulgaris Patients: A Systematic Review and Meta-Analysis

**DOI:** 10.3390/clinpract14030081

**Published:** 2024-05-31

**Authors:** Eleni Paschalidou, Georgios Katsaras, Thomas Papoulakis, Evangelia Kalloniati, Dimitrios Kavvadas, Sofia Karachrysafi, Dorothea Kapoukranidou, Georgios Tagarakis, Theodora Papamitsou

**Affiliations:** 1 Faculty of Medicine, Aristotle University of Thessaloniki, 54124 Thessaloniki, Greece; leni_pasx@hotmail.com (E.P.); tpapoulakis@hotmail.com (T.P.); 2 Department of Paediatrics, General Hospital of Pella—Hospital Unit of Edessa, 58200 Edessa, Greece; gkatsarb@auth.gr; 3 Department of Dermatology, General Hospital of Katerini, 60100 Katerini, Greece; evakalpan@auth.gr; 4 Histology-Embryology Laboratory, Faculty of Health Sciences, Aristotle University of Thessaloniki, 54124 Thessaloniki, Greece; kavvadas@auth.gr (D.K.); skarachry@auth.gr (S.K.); 5 Research Team “Histologistas”, Interinstitutional Postgraduate Program “Health and Environmental Factors”, Department of Medicine, Faculty of Health Sciences, Aristotle University of Thessaloniki, 54124 Thessaloniki, Greece; 6 Department of Physiology, Faculty of Health Sciences, Aristotle University of Thessaloniki, 54124 Thessaloniki, Greece; dkapoukr@auth.gr; 7 Department of Cardiology, AHEPA University Hospital, Faculty of Health Sciences, Aristotle University of Thessaloniki, 54636 Thessaloniki, Greece; gtagarakis@auth.gr

**Keywords:** isotretinoin, acne vulgaris, insulin resistance, adiponectin

## Abstract

Background: Isotretinoin is the drug of choice for severe acne. We sought to examine the potential link between isotretinoin and insulin resistance. Methods: We conducted a systematic review and meta-analysis in accordance with the PRISMA statement. A comprehensive search of the PubMed/MEDLINE, SCOPUS, and Cochrane databases was performed until 12 January 2022 utilizing the PICO (Patient, Intervention, Comparison, Outcome) tool. Fifteen English-language studies focusing on isotretinoin-treated acne patients were included. Serum levels of insulin, glucose, and adiponectin were evaluated before and after treatment, and insulin sensitivity was assessed using the HOMA–IR. A meta-analysis was conducted using RevMan 5.4.1 software, and a quality assessment was undertaken using the ROBINS-I tool. Results: The meta-analysis unveiled a statistically significant rise in the post-treatment levels of adiponectin, an anti-inflammatory agent, which inhibits liver glucose production while enhancing insulin sensitivity (SMD = 0.86; 95% confidence interval (95% CI) = 0.48–1.25, *p*-value < 0.0001; I^2^ = 58%). Our subgroup analysis based on study type yielded consistent findings. However, no statistically significant outcomes were observed for insulin, glucose levels, and the HOMA-IR. Conclusions: There is not a clear association between isotretinoin and insulin resistance, but it appears to enhance the serum levels of adiponectin, which participates in glucose metabolism.

## 1. Introduction

Acne vulgaris (AV) is a chronic multifactorial inflammatory disease of the pilosebaceous units of the skin that affects 80% of adolescents and young adults [1]. The four main factors implicated in the pathogenesis of AV are abnormal follicular desquamation, increased sebum production, Propionibacterium acnes proliferation, and inflammation [2].

Isotretinoin (13-cis-retinoic acid) is a systemic retinoid and Vitamin A (retinol) metabolite which constitutes the only available medication with a potential to be a long-term cure of acne, as it acts on all the pathogenic mechanisms of acne [3].

Although isotretinoin is an effective and relatively well-tolerated medication, many side effects are related to its intake. In the serum of patients treated with isotretinoin, an increase in the levels of total cholesterol and triglycerides and a decrease in the levels of high-density lipoprotein (HDL) are commonly noticed, a phenotype also observed in patients with insulin resistance [4].

Recent studies have shown that adipose tissue, aside from its main function as an energy-storing organ, has immunological and endocrinological functions. The hormones secreted by fat tissue are called adipocytokines, with the main representative of them being adiponectin. Adiponectin is not only an anti-inflammatory agent inhibiting inflammation in a wide range of cell types; it also hinders liver glucose production, increases insulin sensitivity, and contributes to the maintenance of whole body’s energy homeostasis [5].

Previous studies examining the influence of isotretinoin on insulin resistance and serum adiponectin levels in patients with acne vulgaris (AV) have yielded controversial conclusions [6,7]. Therefore, the objective of this meta-analysis is to assess the impact of isotretinoin on glucose metabolism, focusing primarily on changes in insulin resistance and adiponectin levels.

## 2. Materials and Methods

This systematic review adhered to the guidelines outlined in the Preferred Reporting Items for Systematic reviews and Meta-Analyses (PRISMA) statement [8], ensuring consistency with the PRISMA checklist. The review protocol was registered with PROSPERO (International Prospective Register of Systematic Reviews) under the Identifier (ID) Number: CRD42022314953.

### 2.1. Inclusion and Exclusion Criteria

We conducted a search for randomized controlled trials (RCTs), cohort, and case–control studies that compared the levels of adiponectin, insulin, and glucose in the bloodstream, as well as the Homeostatic Model Assessment for Insulin Resistance (HOMA-IR), before and after administering systemic isotretinoin treatment to patients with acne vulgaris. Only studies with fully published texts in English were considered for inclusion.

### 2.2. Search Strategy and Sources

The research approach was crafted according to the Peer Review of Electronic Search Strategies (PRESS) checklist [9], employing both free text and Medical Subject Heading (MeSH) terms along with their synonyms. Search terms such as “isotretinoin”, “acne”, “insulin resistance”, and “adiponectin”, as well as their equivalents, were used. No constraints based on language, location, publication status, or year of publication were imposed (refer to Appendix A, Table A1).

Two reviewers (EP, GNK) independently conducted searches in the following databases: PubMed/Medline, Scopus, and the Cochrane Library. Furthermore, the PROSPERO database was explored for ongoing systematic reviews and meta-analyses (SRMAs). The most recent searches were conducted on 12 January 2022.

### 2.3. Study Selection and Data Extraction

Two reviewers (EP and GNK) selected the studies and extracted data separately. Any discrepancies were resolved through discussion and consensus with a third reviewer (TP). Duplicate references were removed using Mendeley© (version 1.19.8), a reference manager. Data extraction followed predefined forms recommended by the Cochrane Collaboration for Intervention Reviews [10]. In cases where there were questions about study eligibility or the data provided, the authors of the papers were contacted for clarification.

### 2.4. Definitions

Acne vulgaris (AV) is a chronic multifactorial inflammatory condition that affects the pilosebaceous units of the skin [1]. Insulin resistance refers to a clinical condition where the effectiveness of a given amount of insulin, whether produced naturally or administered externally, in enhancing glucose uptake and utilization is diminished compared to that in individuals without such resistance [11]. The HOMA-IR serves as a quantitative measure used to evaluate both insulin resistance and impaired β-cell function’s roles in fasting hyperglycemia by comparing a patient’s fasting glucose levels with the model’s predictions. There are two HOMA-IR formulas, depending on glucose and insulin units. These are as follows: fasting insulin (mU/L) × fasting glucose (nmol/L)/22.5 and HOMA-IR = glucose (mg/dL) × insulin (mU/L)/405 [12].

### 2.5. Risk of Bias Assessment

We utilized the ROBINS-I Cochrane Tool to evaluate bias risk in non-randomized studies [13]. Only studies with a low to moderate risk of bias were considered for inclusion in the quantitative synthesis. For studies identified with a serious or critical risk of bias, a sensitivity analysis was conducted. Visual representations illustrating the bias risk were generated using the Robins tool [14]. The risk of bias assessment was independently carried out by two reviewers (EP and GNK), with any disparities resolved by a third reviewer (TP).

### 2.6. Synthesis

The treatment effects for all outcomes were quantified using mean/median, SD/IQR, and 95% Confidence Intervals (CIs), as they all involved quantitative data. Initially, a comprehensive qualitative synthesis was undertaken. Subsequently, a quantitative synthesis was performed using RevMan (version 5.4.1), wherein various forest plots were generated. Statistical heterogeneity was assessed using the Higgins I^2^ test and Chi-Squared Cochran Q-test (α = 0.1). A high level of statistical heterogeneity was considered when I^2^ exceeded 75%. To account for the heterogeneity among studies, we employed the random-effects model, utilizing the Inverse Variance statistical method with standardized mean difference (SMD) as the effect measure. A sensitivity analysis was performed by excluding studies identified with a serious or critical risk of bias. Additionally, subgroup analyses were conducted based on the type of study. In cases of missing data, efforts were made to contact the authors via email for clarification.

If there were 10 or more studies available for a particular outcome, we conducted an assessment for publication bias. Funnel plots were generated using RevMan 5.4.1 to evaluate this bias.

### 2.7. Quality of Evidence

An evaluation of the quality of evidence for each outcome was independently conducted by two reviewers (EP and GNK) utilizing the GRADE reporting system (Grading of Recommendations Assessment, Development, and Evaluation System) [15]. Any discrepancies between the reviewers were resolved with the assistance of a third reviewer (TP). The evaluation process was facilitated using the online tool GRADEpro GDT [16].

## 3. Results

### 3.1. Qualitative Analysis

#### 3.1.1. Search Results

**Figure 1:**
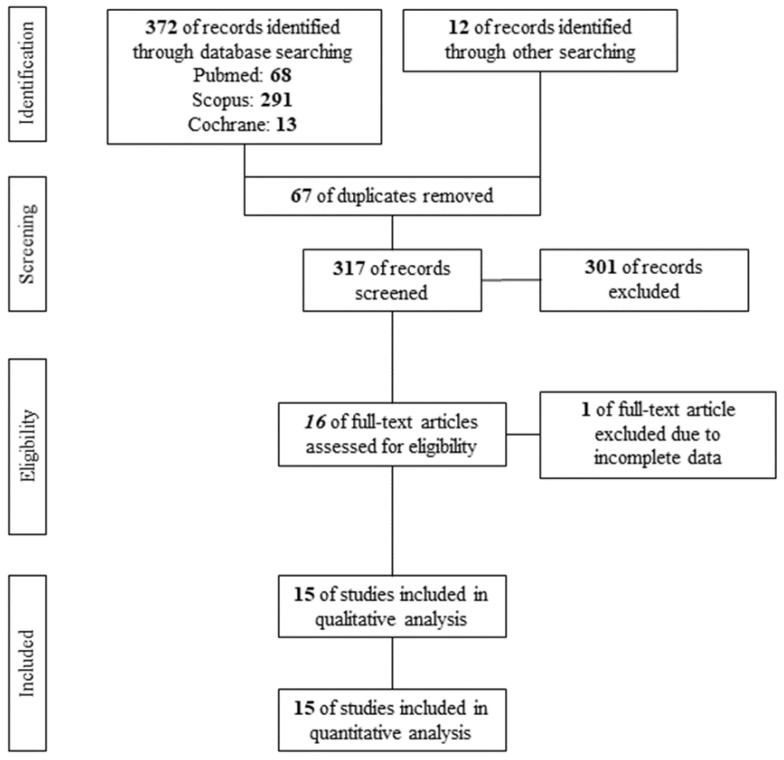
PRISMA flow diagram.

A PRISMA flow diagram of the search results is shown in Figure 1. After the removal of 67 duplicates, 317 studies were screened per Title and Abstract. A total of 16 studies qualified for assessment of eligibility. Finally, 1 study [17] was excluded according to the exclusion criteria, while 15 studies [6,7,18,19,20,21,22,23,24,25,26,27,28,29,30] were found eligible for qualitative and quantitative analysis, including 380 acne vulgaris patients under systemic isotretinoin.

#### 3.1.2. Study Characteristics

Due to the absence of published RCTs, we used nine cohort studies [6,18,19,20,21,22,25,26,28] and six case–control studies [7,23,24,27,29,30] in our systematic review and meta-analysis. The study characteristics are shown in Table 1. All studies were conducted in Asia and Europe. More precisely, 10 studies [6,7,23,24,25,26,27,28,29,30] were conducted in Turkey, 3 studies [19,21,22] in Finland, 1 study [18] in the United Kingdom, and 1 study [20] in Switzerland. Regarding gender, in seven studies [6,7,18,22,24,25,26], the population was mixed; in five studies [23,27,28,29,30], only females were considered, and in three studies [19,20,21], only males were considered. In all studies, isotretinoin was administered orally. In seven studies [7,18,19,20,21,26,30], the dosage of isotretinoin was steady during therapy, with a range between studies of 0.5–1 mg/kg/day, and in one study [29], the dosage was 120–150 mg/kg/day. In five studies [6,23,25,27,28], an increasing dosage was used, while in two studies [22,24], the dosage was not mentioned. In five studies [22,24,25,29,30], the treatment duration was 3 months; in two studies [18,26], it was 4 months; in one study [7], it was 5 months, and in two studies [27,28], it was 6 months. In four studies [6,19,21,23], the treatment duration was dependent to disease progression. In only one study [20], the duration was 5 days, but this study featured a population previously treated with isotretinoin for acne vulgaris.

#### 3.1.3. Risk of Bias Assessment

**Figure A1:**
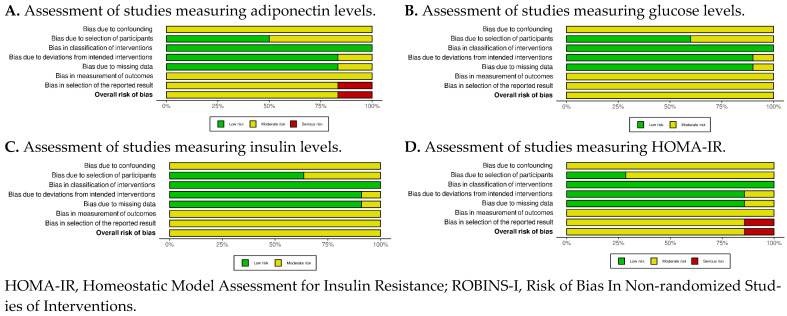
ROBINS-I summary plots of all outcomes.

**Figure A2:**
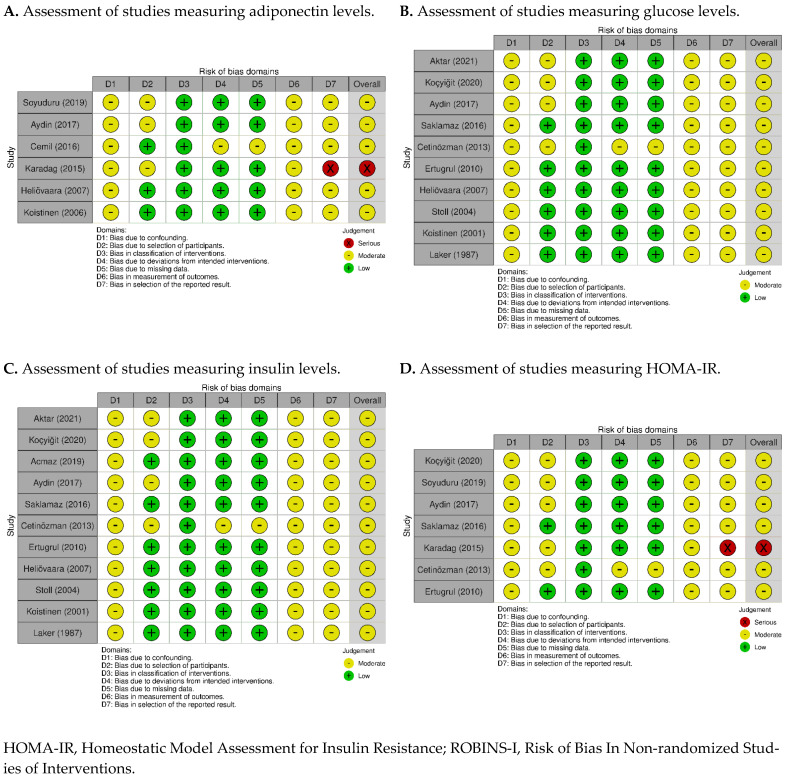
ROBINS-I Traffic Lights Plots of all outcomes [6,18,19,20,22,23,26,27,28,29,30].

For the included observational studies, the results of the risk of bias assessment tool are presented in Appendix A, Figure A1 and Figure A2. Moderate risk of bias was mainly raised in the “Bias due to confounding”, “Bias in measurement of outcomes”, and “Bias in the selection of reported result” domains in almost all studies. Only in a single study [24] was serious risk of bias raised in the “Bias in the selection of reported result” domain, because the authors did not provide all their results.

#### 3.1.4. Outcome Measures

The assessed outcomes of the studies are shown in Table 2. There is great heterogeneity regarding the measurement values of each outcome. Ten studies [6,18,19,20,22,23,26,27,28,30] assessed insulin levels in serum before and after treatment, nine studies [6,18,19,20,22,23,26,27,30] assessed glucose levels in serum before and after treatment, and six [7,21,22,24,25,27] studies assessed adiponectin levels in serum before and after treatment. Finally, six studies [6,7,23,26,27,29] estimated the HOMA-IR before and after treatment, while in five studies [18,19,20,22,30], the latter was calculated by the reviewers EP and GNK.

### 3.2. Quantitative Analysis—Results of Meta-Analysis

#### 3.2.1. Glucose

**Figure A3:**
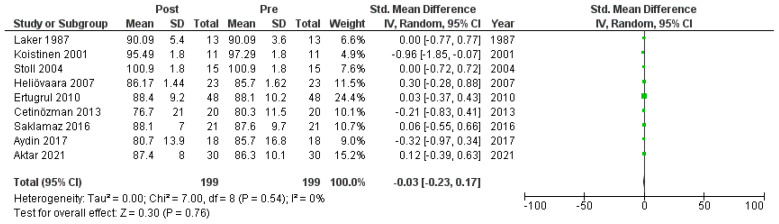
Forest plot: glucose levels before and after treatment with systemic isotretinoin [6,18,19,20,22,23,26,27,30].

**Figure A4:**
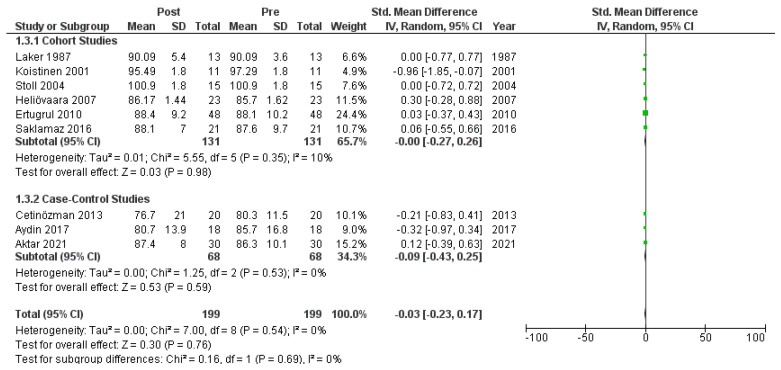
Forest plot (subgroup analysis): glucose levels before and after treatment with systemic isotretinoin [6,18,19,20,22,23,26,27,30].

Nine studies [6,18,19,20,22,23,26,27,30] that assessed glucose levels in serum before and after treatment with systemic isotretinoin were meta-analyzed. No statistically significant difference was found in glucose levels before and after treatment [pooled SMD: −0.03, 95% CI (−0.23–0.17), *p*-value: 0.76; I^2^: 0%] (Appendix A, Figure A3 and Figure A4).

#### 3.2.2. Insulin

**Figure A5:**
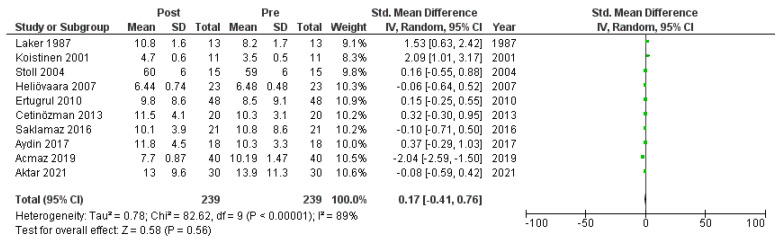
Forest plot: insulin levels before and after treatment with systemic isotretinoin [6,18,19,20,22,23,26,27,30].

**Figure A6:**
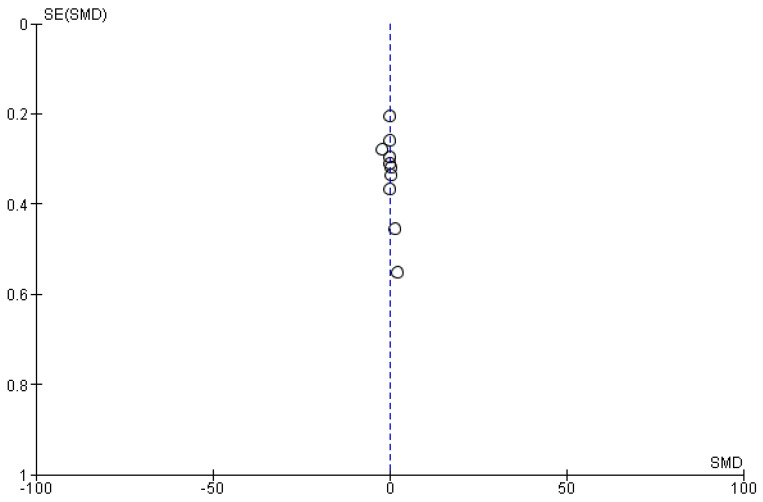
Funnel plot: insulin levels before and after treatment with systemic isotretinoin.

**Figure A7:**
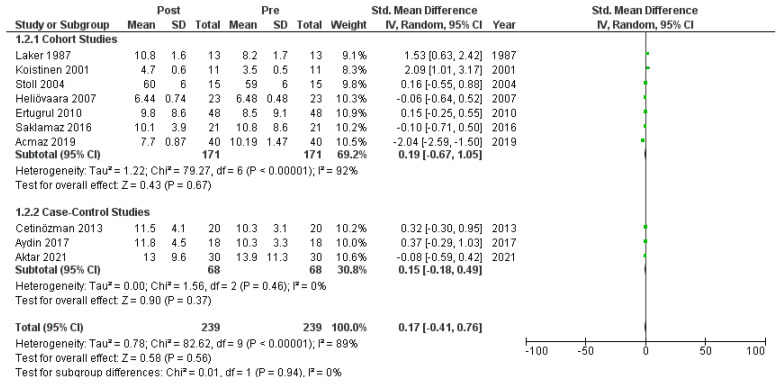
Forest plot (subgroup analysis): insulin levels before and after treatment with systemic isotretinoin [6,18,19,20,22,23,26,27,30].

Ten studies [6,18,19,20,22,23,26,27,28,30] that assessed insulin levels in serum before and after treatment with systemic isotretinoin were meta-analyzed. No statistically significant difference in insulin levels was found before and after treatment [pooled SMD: 0.17, 95% CI (−0.41–0.76), *p*-value: 0.56; I^2^: 89%]. Despite the conducted subgroup analysis, the high statistical heterogeneity remained (I^2^ > 75%), with no statistically significant results (Appendix A, Figure A5, Figure A6 and Figure A7).

#### 3.2.3. Adiponectin

**Figure 2:**
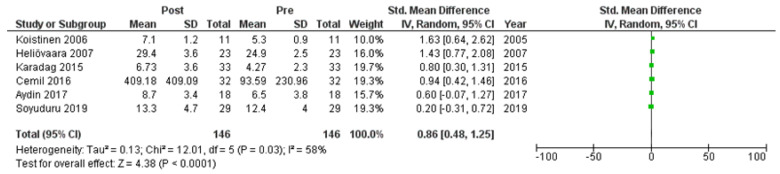
Forest plot: adiponectin levels before and after treatment with systemic isotretinoin [7,21,22,24,25,27].

**Figure 3:**
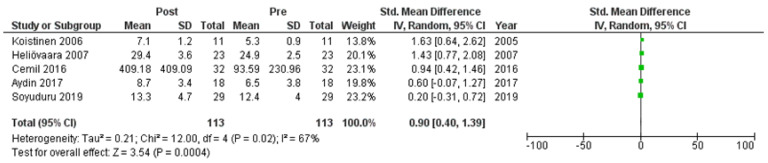
Forest plot (sensitivity analysis): adiponectin levels before and after treatment with systemic isotretinoin [7,21,22,25,27].

**Figure 4:**
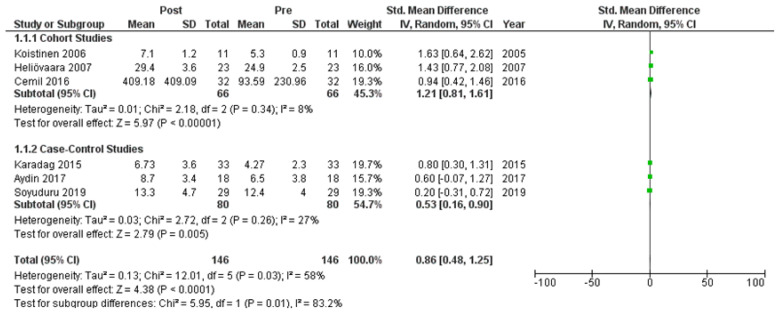
Forest plot (subgroup analysis): adiponectin levels before and after treatment with systemic isotretinoin [7,21,22,24,25,27].

Six studies [7,21,22,24,25,27] assessed adiponectin levels in serum before and after treatment with systemic isotretinoin. Our meta-analysis showed that adiponectin increases significantly after treatment [pooled SMD: 0.86, 95% CI (0.48−1.25), *p*-value < 0.0001; I^2^: 58%] (Figure 2). We conducted a sensitivity analysis, excluding one study [24] that was assessed as having a serious risk of bias, with similar results [pooled SMD: 0.90, 95% CI (0.40−1.39), *p*-value: 0.0004; I^2^: 67%] (Figure 3), but in our subgroup analysis, the meta-analyzed cohort studies revealed a higher and more statistically significant increase in adiponectin levels after treatment [pooled SMD: 1.21, 95% CI (0.81−1.61), *p*-value < 0.00001; I^2^: 8%] (Figure 4). Two of the meta-analyzed studies [21,22] measured the levels of adiponectin 1–3 months after treatment and showed that even though isotretinoin increases adiponectin levels, this increase is transient.

#### 3.2.4. HOMA-IR

**Figure A8:**
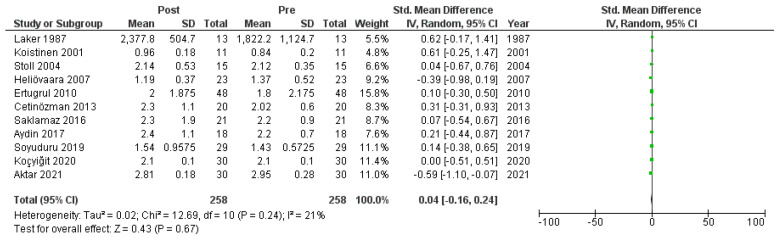
Forest plot: HOMA-IR before and after treatment with systemic isotretinoin [6,7,18,19,20,22,23,26,27,29,30].

**Figure A9:**
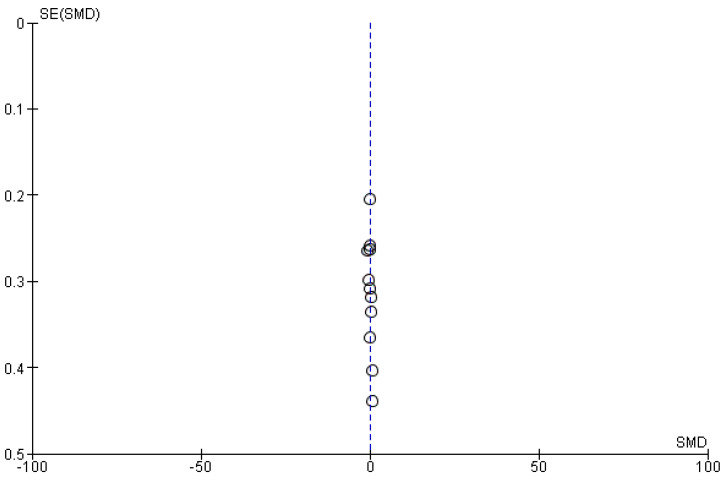
Funnel plot: HOMA−IR before and after treatment with systemic isotretinoin.

**Figure A10:**
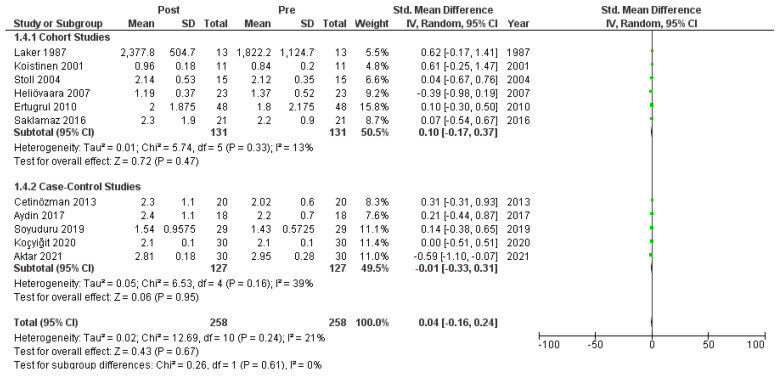
Forest plot (subgroup analysis): HOMA-IR before and after treatment with systemic isotretinoin [6,7,18,19,20,22,23,26,27,29,30].

We meta-analyzed the HOMA-IR before and after treatment with systemic isotretinoin from 11 studies [6,7,18,19,20,22,23,26,27,29,30]. No statistically significant difference was found in the HOMA-IR before and after treatment [pooled SMD: 0.04, 95% CI (−0.16–0.24), *p*-value: 0.67; I^2^: 21%]. Despite the conducted subgroup analysis, not one statistically significant result was found (Appendix A, Figure A8, Figure A9 and Figure A10).

### 3.3. Strength of Evidence GRADE Reporting System

The results of the quality of evidence assessment regarding the comparison of insulin, glucose, and adiponectin levels, as well as the HOMA-IR, before and after treatment with systemic isotretinoin are shown in Appendix A, Table A2. Adiponectin, insulin, and glucose levels were judged to be of “High” strength of evidence, while the HOMA-IR was judged to be of “Moderate” strength of evidence.

## 4. Discussion

To the best of our knowledge, Tsai et al. [31] conducted the only systematic review and meta-analysis available in the literature regarding the effect of isotretinoin treatment on glucose metabolism in patients with acne. They concluded that treating acne patients with isotretinoin does not substantially change the HOMA-IR values but significantly increases the serum adiponectin level. In our updated systematic review and meta-analysis, we included three more subsequently published studies, and our results were consistent with the ones of Tsai et al. What is noteworthy is that, despite the fact that, in four [21,24,25,27] of the five meta-analyzed studies, the means of the post-treatment adiponectin values differed in a statistically significant manner from those of the pre-treatment measurements, the means and the reference ranges of all five studies [7,21,24,25,27] were inside the normal values, which are 0–30 μg/mL [32]. These findings have pathophysiological value and, indirectly, clinical value; the increase might be significant pathophysiologically, but there is no major clinical outcome.

Despite the above results, a possible increase in serum glucose in patients receiving isotretinoin is still under investigation. The European Medicines Agency states that patients with diabetes, obesity, alcoholism, or dyslipidemia treated with isotretinoin may require more frequent monitoring of serum lipids and/or blood glucose levels [3]. Namely, elevated fasting blood sugars have been recorded and new cases of diabetes have been identified while on isotretinoin medication. Santos-Pérez et al. [33] reported the onset of type 1 diabetes mellitus in a 17-year-old patient receiving six months of isotretinoin treatment without a family history of diabetes. Anti-glutamate decarboxylase 65 (GADA), anti-islet cell (ICA), anti-insulin (IAA), and anti-tyrosine phosphatase (anti-IA2) antibodies were requested throughout the diagnostic procedure, all of which were negative, indicating that the pancreatic beta cell were destroyed by non-autoimmune processes. Dicembrini et al. [34] published the case of a 28-year-old man who was diagnosed with latent autoimmune diabetes after being treated with isotretinoin. The above-reported cases, although rare, raise concerns about the molecular mechanisms of action of isotretinoin.

All-trans retinoic acid (ATRA), the result of 13-cis-retinoic acid isomerization by sebocytes, changes gene expression by binding to and activating the retinoic acid receptors (RARs) [35]. In human keratinocytes, both the expression of p53 and proapoptotic caspases are increased by ATRA, which is also responsible for the apoptosis of the former. Furthermore, neutrophil apoptosis caused by ATRA and p53 possibly minimizes inflammation in acne. During treatment, isotretinoin induces the death of sebocytes and consequently reduces sebum production, while the microscopic image of it is the involution of sebaceous glands [36]. In those glands, nuclear levels of Forkhead box protein O1 and O3 (FoxO1, FoxO3) are increased by ATRA as well, further reducing sebum production [37].

Although the exact mechanisms behind the regulation of fluctuations in adiponectin levels in plasma and cells are yet to be revealed, recent studies’ results lean towards the possibility that adiponectin is controlled during transcription and post transcription. Peroxisome proliferator-activated receptor-g (PPARγ), CCAAT/enhancer-binding protein, and FoxO1 appear to be transcription factors that increase adiponectin expression, while agonists of the nuclear receptor and PPARγ also increase its multimerization and secretion [38]. It has also been shown that the activation of the latter not only multiplies small, insulin-sensitive adipocytes by facilitating the process of their creation but increases the response of adipose-derived hormone adiponectin as well [39]. On the other hand, FoxO1, one of the Forkhead box O transcription factors, participates in the adjustment of adipocyte differentiation. More specifically, even though FoxO1 seems to upregulate adiponectin transcription, it also appears to suppress PPARγ gene expression and its interaction with CCAAT/enhancer-binding protein α obscurely increases adiponectin gene transcription [40,41].

Laboratory results were contradictory. To begin with, Landrier et al. [42] observed a decreased expression of adiponectin in white muscle adipose tissue amidst the consumption of a diet high in Vitamin A, while Kovács et al. [27] reported that isotretinoin treatment decreases adiponectin mRNA expression in human sebaceous cells. According to Kalisz et al. [43], treatment with all-trans retinoic acid increases both the synthesis and secretion of adiponectin by perivascular adipose tissue in apolipoprotein E-deficient mice, significantly increasing its levels in visceral adipose tissue. It is possible that adiponectin is secreted by different cell types, and its levels in sebaceous cells do not represent the ones measured in serum in clinical practice. In addition, the post-isotretinoin treatment adiponectin increase in acne patients may be triggered by the anti-inflammatory mechanisms of isotretinoin. More research is needed to clarify these mechanisms and associate scientific findings and clinical measurements.

Adipocytes of a growing adipose tissue are the first to develop insulin resistance, while ectopic fat storage in organs such as the liver and muscles as a result of its unsuccessful deposition in the adipose tissue is considered to be the spread mechanism of such resistance in those organs. The above-mentioned ectopic lipid storage appears to be controlled by usual genetic mutations as well [44].

Not long ago, ApoC3 polymorphisms were associated with the lean male population’s susceptibility to NAFLD and insulin resistance leading to a rise in ApoC3 plasma levels by approximately 30% and postprandial hypertriglyceridemia caused by ApoC3’s altering effect on lipoprotein lipase activity. It was also found that this alteration consequently increased the amount of chylomicron remnants stored in the liver. In addition, an increased hepatic triacylglycerol (TAG)/DAG concentration was observed in transgenic mice on a high-fat diet that overexpressed human ApoC3 in the liver due to the activation of hepatic PKCε (Protein Kinase Cε) and the development of hepatic insulin resistance [45]. The isomerization of isotretinoin to ATRA takes place inside human sebaceous cells, increasing nuclear levels of FoxO1 [35,37]. This particular protein raises apolipoprotein C3 levels, which subsequently favorizes the storage of very-low-density lipoprotein (VLDL) over lipids into cells, causing hypertriglyceridemia [46]. The lipid profiles of patients receiving isotretinoin treatment and those with insulin resistance were found to share the same disorders. More specifically, an increase in triglycerides and decrease in high-density lipoprotein (HDL-C) were the most common laboratory findings. Finally, although FoxO1 plays a major role in the insulin signaling pathway, not much is known about its association with insulin resistance in adipocytes, the most critical cell type in developing it [43,47].

### Limitations

Finally, it is important to address the limitations encountered in our study. Firstly, the absence of randomized clinical trials in the literature compelled us to rely solely on cohort and case–control studies available in English. This approach may have excluded relevant articles published in other languages. Secondly, the limited number of studies and the predominantly Turkish and Finnish populations studied may limit the generalizability of our findings. Thirdly, the included studies exhibited variations in the baseline characteristics of the study population (such as sex, BMI, age, and comorbidities) and methodology (including dosage and duration of treatment), which could have impacted adiponectin and insulin resistance levels. Despite efforts to account for these variations, they remain potential confounding factors. However, it is worth noting that the I2 test indicated low heterogeneity, which strengthens the reliability of our results.

It appears that relatively little attention is given to exploring the relationship between isotretinoin and insulin resistance. This observation is supported by the small number of published studies on the topic and the absence of any registered randomized controlled trials specifically investigating the potential role of isotretinoin in affecting insulin resistance. Despite the existence of a few registered RCTs examining the safety and efficacy of isotretinoin, none of them appear to include an evaluation of its impact on insulin resistance.

## 5. Conclusions

In summary, the systematic review and meta-analysis indicate that isotretinoin treatment in acne patients is associated with a notable elevation in serum adiponectin levels. However, the evidence suggests that isotretinoin does not have a significant effect on altering insulin resistance, as measured by the HOMA-IR.

## Figures and Tables

**Table 1:** Study characteristics.

Author (Year)	Country	Study Design	N of Acne Patients	Age (Years)	Oral Isotretinoin Dosage	Treatment Duration (Months)	Control	Parameters Assessed
Laker (1987) [18]	U.K.	Cohort	13 (M:F = 10:3)	13–32 *	1.0 mg/kg/day	4	no	glucose, insulin
Koistinen (2001) [19]	Finland	Cohort	11 (M:F = 11:0)	24 (2) ^	∼0.5 mg/kg/day	4–6	no	glucose, HbA1c%, insulin
Stoll (2004) [20]	Switzerland	Cohort	15 (M:F = 15:0)	28.3 (1.7) ^	1.0 mg/kg/day	5 days ^1^	no	glucose, insulin
Koistinen (2006) [21]	Finland	Cohort	11 (M:F = 11:0)	24 (2) ^	∼0.5 mg/kg/day	5 (6–10) *	no	adiponectin
Heliövaara (2007) [22]	Finland	Cohort	23 (M:F = 12:11)	24.9 (0.9) ^	N/A	3	no	adiponectin, glucose, insulin
Ertugrul (2010) [6]	Turkey	Cohort	48 (M:F = 13:35)	22 (18–38) **	0.5–0.75 mg/kg/day, adjusted to 0.88 mg/kg/day 1 month after	≥4	no	glucose, insulin, HOMA-IR
Cetinözman (2013) [23]	Turkey	Case–control	26 (M:F = 0:26)	24.7 (3.9) ^	20 mg/day increased to max. 50 mg/day	7.5 (6–10) *	yes	glucose/insulin, HOMA-IR
Karadag (2015) [24]	Turkey	Case–control	33 (M:F = 15:18)	19.8 (4.1) ^	N/A	3	yes	adiponectin, glucose, insulin
Cemil (2016) [25]	Turkey	Cohort	32 (M:F = 20:12)	18.9 (2.57) ^	0.5–0.6 mg/kg/day adjusted to 0.6–0.75 mg/kg/day after 1 month	3	no	adiponectin
Saklamaz (2016) [26]	Turkey	Cohort	21 (M:F = 6:15)	23.0 (4.1) ^	0.5–0.8 mg/kg/day	4	no	glucose, insulin, HOMA-IR
Aydin (2017) [27]	Turkey	Case–control	18 (M:F = 0:18)	23.4 (3.4) ^	20 mg/day increased to max. 50 mg/day	6	yes	adiponectin, glucose, insulin, HOMA-IR
Soyuduru (2019) [7]	Turkey	Case–control	29 (M:F = 15:14)	20.5 (1.9) ^	0.5 mg/kg/day	5	yes	adiponectin, HOMA-IR
Acmaz (2019) [28]	Turkey	Cohort	40 (M:F = 0:40)	18–40 *	20 mg/day increased to max. 40 mg/day	6	no	insulin
Koçyiğit (2020) [29]	Turkey	Case–control	30 (M:F = 0:30)	23.2 (3.7) ^	120–150 mg/kg/day	3	yes	glucose, insulin, HOMA-IR
Aktar (2021) [30]	Turkey	Case–control	30 (M:F = 0:30)	22.1 (3.4) ^	0.5 mg/kg/day	3	yes	glucose, insulin

Abbreviations: F, females; HOMA-IR, Homeostatic Model Assessment for Insulin Resistance; M, males; N/A, not available. * range. ^ mean (SD). ** median (IQR). ^1^ previous treatment with isotretinoin on average 5 years earlier (3–10).

**Table 2:** Outcome measurements of all included studies.

	HOMA-IR		Adiponectin		Glucose		Insulin	
Author (Year)	Pre-Treatment	Post-Treatment	Pre-Treatment	Post-Treatment	Pre-Treatment	Post-Treatment	Pre-Treatment	Post-Treatment
Laker (1987) [18]	1822.2 (1124.7) ^	2377.8 (504.7) ^	N/A	N/A	5 (0.2) mmol/L	5 (0.3) mmol/L	8.2 (1.7) IU/L	10.8 (1.6) IU/L
Koistinen (2001) [19]	0.84 (0.2) ^	0.96 (0.18) ^	N/A	N/A	5.4 (0.1) mmol/L	5.3 (0.1) mmol/L	3.5 (0.5) mIU/L	4.7 (0.6) mIU/L
Stoll (2004) [20]	2.12 (0.35) ^	2.14 (0.53) ^	N/A	N/A	5.6 (0.1) mmol/L	5.6 (0.1) mmol/L	59 (6) pmol/L	60 (6) pmol/L
Koistinen (2006) [21]	N/A	N/A	5.3 (0.9) μg/mL	7.1 (1.2) μg/mL	N/A	N/A	N/A	N/A
Heliövaara (2007) [22]	1.37 (0.52) ^	1.19 (0.37) ^	24.9 (2.5) μg/mL	29.4 (3.6) μg/mL	85.70 (1.62) mg/dL	86.17 (1.44) mg/dL	6.48 (0.48) mIU/L	6.44 (0.74) mIU/L
Ertugrul (2010) [6]	1.8 (2.175)	2.0 (1.875)	N/A	N/A	88.1 (10.2) mg/dL	88.4 (9.2) mg/dL	8.5 (9.1) μIU/mL	9.8 (8.6) μIU/mL
Cetinözman (2013) [23]	2.02 (0.6)	2.3 (1.1)	N/A	N/A	80.3 (11.5) mg/dL	76.7 (21.0) mg/dL	10.3 (3.1) μIU/mL	11.5 (4.1) μIU/mL
Karadag (2015) [24]	N/A	N/A	4.27 (2.30) ng/mL	6.73 (3.60) ng/mL	N/A	N/A	N/A	N/A
Cemil (2016) [25]	N/A	N/A	93.59 (230.96) μg/L	409.18 (409.09) μg/L	N/A	N/A	N/A	N/A
Saklamaz (2016) [26]	2.2 (0.9)	2.3 (1.9)	Ν/A	Ν/A	87.6 (9.7) mg/dL	88.1 (7.0) mg/dL	10.8 (8.6) μIU/mL	10.1 (3.9) μIU/mL
Aydin (2017) [27]	2.2 (0.7)	2.4 (1.1)	6.5 (3.8) μg/mL	8.7 (3.4) μg/mL	85.7 (16.8) mg/dL	80.7 (13.9) mg/dL	10.3 (3.3) μIU/mL	11.8 (4.5) μIU/mL
Soyuduru (2019) [7]	1.43 (0.5725)	1.54 (0.9575)	12.4 (4.0) μg/mL	13.3 (4.7) μg/mL	N/A	N/A	N/A	N/A
Acmaz (2019) [28]	N/A	N/A	N/A	N/A	N/A	N/A	10.19 (1.47) *	7.70 (0.87) *
Koçyiğit (2020) [29]	2.1 (0.1)	2.1 (0.1)	N/A	N/A	82.6 (7.03) mg/dL	N/A	10.1 (4.8) μIU/mL	N/A
Aktar (2021) [30]	2.95 (0.28) ^	2.81 (0.18) ^	N/A	N/A	86.3 (10.1) mg/dL	87.4 (8.0) mg/dL	13.9 (11.3) μIU/mL	13.0 (9.6) μIU/mL

Abbreviations: HOMA-IR, Homeostatic Model Assessment for Insulin Resistance; N/A, not available; SD, Standard Deviation. All results are presented in means (SD). ^ calculated by the reviewers. * the authors did not provide the measurement.

## Data Availability

All data produced or examined throughout this study have been incorporated into this published article.

## Appendix A

**Table A1:** Search strategy per database.

Database	Search String
Pubmed/Medline	((isotretinoin [Title/Abstract]) OR (“13-cis-Retinoic Acid” [Title/Abstract]) OR (Accutane [Title/Abstract]) OR (Roaccutane [Title/Abstract]) AND (english [Filter])) AND ((insulin [Title/Abstract]) OR (glucose [Title/Abstract]) OR (hyperglycemia [Title/Abstract]) OR (diabetes [Title/Abstract]) OR (“diabetes mellitus” [Title/Abstract]) OR (“insulin resistance” [Title/Abstract]) OR (“glucose intolerance” [Title/Abstract]) OR (adiponectin [Title/Abstract]) OR (adipokine [Title/Abstract]) OR (“apM-1 Protein” [Title/Abstract]) OR (“ACRP30 Protein” [Title/Abstract]) OR (Adipocyte [Title/Abstract]) AND (English [Filter]))
Scopus	TITLE-ABS-KEY (((isotretinoin) OR (“13-cis-Retinoic Acid”) OR (accutane) OR (roaccutane)) AND ((insulin) OR (glucose) OR (hyperglycemia) OR (diabetes) OR (“diabetes mellitus”) OR (“insulin resistance”) OR (“glucose intolerance”) OR (adiponectin) OR (adipokine) OR (“apM-1 Protein”) OR (“ACRP30 Protein”) OR (adipocyte))) AND (LIMIT-TO (DOCTYPE, “ar”)) AND (LIMIT-TO (LANGUAGE, “English”))
Cochrane Library	#1: isotretinoin OR “13 cis Retinoic Acid” OR Accutane OR Roaccutane #2: insulin OR glucose OR hyperglycemia OR diabetes OR “diabetes mellitus” OR “insulin resistance” OR “glucose intolerance” OR adiponectin OR adipokine OR “apM 1 Protein” OR “ACRP30 Protein” OR Adipocyte #3: #1 AND #2. Final results in the Trials field was 13.

**Table A2:** GRADE summary of findings in the effect estimates of the outcomes regarding the effect of isotretinoin on insulin resistance and serum adiponectin levels in acne vulgaris patients.

Outcomes	Pooled Effects (95% CI)	№ of Participants (Studies)	Certainty of the Evidence (GRADE)	Comments
	Risk with Systematic Isotretinoin			
Insulin	SMD 0.17 SD higher (0.41 lower to 0.76 higher)	239 (10 observational studies)	⨁⨁⨁⨁ High	Systematic isotretinoin therapy results in little-to-no difference in insulin.
Glucose	SMD 0.03 SD lower (0.23 lower to 0.17 higher)	199 (9 observational studies)	⨁⨁⨁⨁ High	Systematic isotretinoin therapy results in little-to-no difference in glucose.
Adiponectin	SMD 0.86 SD higher (0.48 higher to 1.26 higher)	146 (6 observational studies)	⨁⨁⨁⨁ High	Systematic isotretinoin therapy increases adiponectin.
HOMA-IR	SMD 0.04 SD higher (0.16 lower to 0.24 higher)	258 (11 observational studies)	⨁⨁⨁◯ Moderate	Systematic isotretinoin therapy results in little-to-no difference in HOMA-IR.
CI: confidence interval; SMD: standardized mean difference.				
GRADE Working Group grades of evidence. High certainty: We are very confident that the true effect lies close to that of the estimate of the effect. Moderate certainty: We are moderately confident in the effect estimate: the true effect is likely to be close to the estimate of the effect, but there is a possibility that it is substantially different. Low certainty: Our confidence in the effect estimate is limited: the true effect may be substantially different from the estimate of the effect. Very low certainty: We have very little confidence in the effect estimate: the true effect is likely to be substantially different from the estimate of effect.				
